# Children’s Quality of Life Based on the KIDSCREEN-27: Child Self-Report, Parent Ratings and Child-Parent Agreement in a Swedish Random Population Sample

**DOI:** 10.1371/journal.pone.0150545

**Published:** 2016-03-09

**Authors:** Anne H. Berman, Bojing Liu, Sara Ullman, Isabel Jadbäck, Karin Engström

**Affiliations:** 1 Department of Clinical Neuroscience, Center for Psychiatric Research, Karolinska Institutet, Stockholm, Sweden; 2 Department of Psychology, Uppsala University, Uppsala, Sweden; 3 Department of Public Health Sciences, Karolinska Institutet, Stockholm, Sweden; University of Bath, UNITED KINGDOM

## Abstract

**Background:**

The KIDSCREEN-27 is a measure of child and adolescent quality of life (QoL), with excellent psychometric properties, available in child-report and parent-rating versions in 38 languages. This study provides child-reported and parent-rated norms for the KIDSCREEN-27 among Swedish 11–16 year-olds, as well as child-parent agreement. Sociodemographic correlates of self-reported wellbeing and parent-rated wellbeing were also measured.

**Methods:**

A random population sample consisting of 600 children aged 11–16, 100 per age group and one of their parents (N = 1200), were approached for response to self-reported and parent-rated versions of the KIDSCREEN-27. Parents were also asked about their education, employment status and their own QoL based on the 26-item WHOQOL-Bref. Based on the final sampling pool of 1158 persons, a 34.8% response rate of 403 individuals was obtained, including 175 child-parent pairs, 27 child singleton responders and 26 parent singletons. Gender and age differences for parent ratings and child-reported data were analyzed using *t-*tests and the Mann-Whitney *U*-test. Post-hoc Dunn tests were conducted for pairwise comparisons when the p-value for specific subscales was 0.05 or lower. Child-parent agreement was tested item-by-item, using the Prevalence- and Bias-Adjusted Kappa (PABAK) coefficient for ordinal data (PABAK-OS); dimensional and total score agreement was evaluated based on dichotomous cut-offs for lower well-being, using the PABAK and total, continuous scores were evaluated using Bland-Altman plots.

**Results:**

Compared to European norms, Swedish children in this sample scored lower on Physical wellbeing (48.8 SE/49.94 EU) but higher on the other KIDSCREEN-27 dimensions: Psychological wellbeing (53.4/49.77), Parent relations and autonomy (55.1/49.99), Social Support and peers (54.1/49.94) and School (55.8/50.01). Older children self-reported lower wellbeing than younger children. No significant self-reported gender differences occurred and parent ratings showed no gender or age differences. Item-by-item child-parent agreement was slight for 14 items (51.9%), fair for 12 items (44.4%), and less than chance for one item (3.7%), but agreement on all dimensions as well as the total score was substantial according to the PABAK-OS. Visual interpretation of the Bland-Altman plot suggested that when children’s average wellbeing score was lower parents seemed to rate their children as having relatively higher total wellbeing, but as children’s average wellbeing score increased, parents tended to rate their children as having relatively lower total wellbeing. Children living with both parents had higher wellbeing than those who lived with only one parent.

**Conclusions:**

Results agreed with European findings that adolescent wellbeing decreases with age but contrasted with some prior Swedish research identifying better wellbeing for boys on all dimensions but Social support and peers. The study suggests the importance of considering children’s own reports and not only parental or other informant ratings. Future research should be conducted at regular intervals and encompass larger samples.

## Introduction

Systematic measurement of child and adolescent mental health on a public health basis has not been implemented anywhere in the world [1]. Such measurement is important for estimating population needs for mental health services, not fully met in any country, but estimated at about 5–20% of the population under 18 [2]. Measuring symptoms of mental ill-health and everyday emotional and behavioral difficulties is one way to evaluate the extent of problematic mental health among children and adolescents. An alternative, positively oriented method is to assess quality of life (QoL) as an indirect evaluation of mental health status.

The QoL concept covers a wide scope and is generally divided into health-related quality of life measures (HRQOL), which are illness-related and symptom-focused, or pure QoL measures, covering a variety of dimensions in human wellbeing. QoL measures for children contribute information on everyday functioning in home and school contexts and can thus add significant information beyond symptom-focused measures. Measuring quality of life can thus serve as part of a strategy to evaluate the effects of contextual burdens such as negative sociodemographic indicators like poverty, poor schooling or, for example, illness in a sibling or a parent, as well a child’s own illness or illness-specific treatment outcomes. To facilitate international comparisons of children’s QoL, both on the population level *and* in health-related contexts, a standardized global measure of wellbeing called the KIDSCREEN measure was developed by a European consortium and is now available in 38 languages, in 10-, 27- and 52-item versions [3].

The KIDSCREEN has been used in at least 50 studies so far, mostly in Europe but also in Africa, Asia and South America. Three major international studies have incorporated KIDSCREEN, using the child self-report version of the KIDSCREEN-10 among school-aged children [4], the parent-rating version of the KIDSCREEN-10 evaluating children’s mental health and well-being in 27 EU countries [5], and the self-report version in a sample of children with cerebral palsy who filled in the KIDSCREEN-52 on their own or with parental help [6]. Findings suggest that the KIDSCREEN questionnaires, systematically developed to measure equivalent dimensions in wellbeing across a variety of cultures, accurately estimate cross-national differences between children since differences due to measurement problems have been minimized [7].

None of the KIDSCREEN studies published have systematically reported child self-reported norms and parental rating norms, nor have they assessed child-parent agreement. Parental proxy measures have frequently been used to assess children’s mental health and wellbeing, since children have been considered as lacking cognitive and language skills for self-report of these constructs. However, recent evidence suggests that children’s self-reports can be reliable and valid, and that differences between children’s and parents’ assessments of well-being may stem more from general issues related to proxy assessment of another individual’s state of being, rather than relating to children’s lesser cognitive or linguistic skills [8]. Comparisons of parent ratings and child self-report on specific measures significantly contribute to knowledge about the extent of child-parent agreement and the direction of any differences identified. This can be a crucial matter if parents are asked to make decisions regarding their children’s health or well-being, and the children’s own self-report differs from parental ratings [8]. Research on child-parent agreement has, however, shown mixed findings, with some studies finding high child-parent agreement and some low [8, 9], clearly warranting further research.

Three prior Swedish studies have reported KIDSCREEN child self-report data, all based on the KIDSCREEN-52 [10]. One study measured wellbeing among 10 000 12–15 year olds in the 6^th^ and 9^th^ grades [11], another reported data from a sample of 1229 6^th^ and 8^th^ graders [12]; and a third country-wide study mapped mental health among 172 298 children and adolescents in the 6^th^ and 9^th^ grades [13]. While contributing valuable information on children’s wellbeing and mental health status based on KIDSCREEN, Swedish research has been limited in that children’s ages were restricted to the 12- and 15-year age groups, no norm data were reported and no sociodemographic indicators were assessed. Furthermore, no collateral data from parents or teachers were collected.

The current study addresses the need for concurrent norms for children and their parents, as well as assessing child-parent agreement. Since prior research on the KIDSCREEN has not contained data in this particular configuration, the study has international relevance although its focus is a Swedish population sample. Our primary objective was to provide child self-report and parent-rating norms for the KIDSCREEN-27 [14, 15], by gender and age for 11 to 16 year olds. Secondary objectives were to analyze child-parent agreement and to assess associations between sociodemographic correlates and norms, as well as sociodemographic associations with child-parent agreement. The underlying clinical and public health objectives of the study were to facilitate assessment of future respondents’ scores in comparison to the norm data presented herein.

## Methods

Data from the present cross-sectional study were gathered between June 13 and December 31, 2012. Ethical approval was granted by the Stockholm Regional Ethical Vetting Board at Karolinska Institutet (No. 2012/688-31/5). Parents granted informed consent for themselves and for their children under 15 to participate in this research; children 15 and older gave their own consent to participate in the research, as stipulated in The Act concerning the Ethical Review of Research Involving Humans (SFS 2003:460). Children under 15 also gave their informed consent in addition to parental consent, although this is not required by law. Both parents and children granted written informed consent before responding to the study questionnaires, either online or on paper.

### Participants and procedure

A random sample was drawn on May 30, 2012 from the Swedish tax authority registry of child parent/guardians with children 11 to 16 years old. The main home address was provided for each child. Six hundred children and adolescents (*n* = 100 for each age group) and one of their parents (unspecified regarding which parent) were approached by postal mail. Information was sent out on four occasions: one letter and two reminders with links to online versions of the questionnaires, and a third and final reminder including paper versions of the questionnaires. Each letter included a serial number for the family, to enable pairing of children and parents from the same household. Respondents to the online questionnaires, uploaded on the SurveyXact secure online questionnaire system [16], gave their informed consent and entered their serial number online. Data were stored in a secure, encrypted database available only to the research team.

The first letter was sent by post in the middle of June 2012, and reminders were sent two and seven weeks later. A third reminder including paper questionnaires was sent nine weeks after the first letter. All questionnaire responses before December 31, 2012 were included in the data set analyzed. No compensation was offered to participants. See Table 1 for participant flow and study response rates.

**Table 1:** Participant flow, questionnaire formats and response rates for the study.

	Format online/paper	Children only	Parent only	Both children and parents
Initial sampling pool		600	600	1200
Non-participants who explained their lack of participation^a^		21	21	42
Final sampling pool		579	579	1158
Cumulative n				
Response without reminder	online	43	46	89
After 1 reminder	online	89	109	198
After 2nd & 3^rd^ reminders	online	127	147	274
After 3rd reminder (including paper version)^b^	paper	202	201	403
Response rate		34.8%	34.7%	34.8%

a. Non-participants included here had incorrect addresses or explicitly communicated their reasons for not participating as difficulties with Swedish language, having a disabled child, and lacking interest in participating in the study. The remaining non-participants (377 children and 378 parents) simply did not respond to the researchers’ letters.

b. Of the total questionnaires received from parents and children, 175 were from *matched* child-parent pairs (29.2%) with an additional 27 from only the child and 26 from only the parent.

A data set of n = 403 (34.8%) was obtained. Of the 403 questionnaires received online and by post, 350 (30.2% of the total sample; 86.8% of all respondents) were answered by 175 child-parent pairs, while 27 (6.6% of all respondents) were answered by children only, and 26 (6.5%) were answered by parents only. The percentage of participants answering the questionnaires online was 66.2% and in paper versions (by post) was 33.8%.

Table 2 shows sample characteristics. The children’s mean age was 13.7 years (SD = 1.83), with 39.4% boys. The mean parental age was 45.6 years (SD = 5.49), with 18.9% males. A large majority of the participants were born in Sweden (88.1% of parents; 93.5% of children). Most of the children lived with both parents (80.3%), and most of the parents lived with a partner (86.1%). Regarding employment and education, almost all of the parents were employed; fewer than one-third had 11 years or less of education, and 40% had 15 years or more of education. The mean parental score for each domain of WHOQOL-BREF (scale 0–100; standard deviations [SD] in parentheses) was for Physical Health 75.4 (18.82); Psychological Health 72.8 (16.13); Social relationships 69.4 (17.34); and Environment 73.5 (14.16).

**Table 2:** Sample description, N = 403^a^.

Sociodemographic and psychosocial factors	Children N (%)	Parents N (%)
Mean age (SD) ^b^	13.7(1.83)	45.6(5.49)
Sex		
Male	78 (39.4)	38 (18.9)
Female	120 (60.6)	163 (81.1)
Born in Sweden		
Yes	188(93.5)	177 (88.1)
No	13(6.5)	24 (11.9)
Child living status		
with both parents	159 (80.3)	
with either parent/others	39 (19.7)	-
Parent living status		
with partner	-	173 (86.1)
without partner	-	28 (13.9)
Employment		
Employed		172 (86.0)
Other		28 (14.0)
Education		
11 years or less	-	56 (28.0)
12–14 years	-	64 (32.0)
15 years or more	-	80 (40.0)
WHOQOL-BREF, scale 0–100		mean (*SD*)
Physical Health	-	75.4 (18.82)
Psychological Health	-	72.8 (16.13)
Social relationships	-	69.4 (17.34)
Environment	-	73.5 (14.16)
Total	N = 202	N = 201

a. Both parent and child responded, N = 175; Parent only responded N = 26; Child only responded, N = 27.

b. The children approached were 11–16 years old but some had turned 17 by the time they answered the questionnaire.

### Instruments

#### Children and adolescents’ quality of life (KIDSCREEN-27)

We chose to use the KIDSCREEN-27 rather than the KIDSCREEN-52 because of its excellent psychometric properties and in order to reduce the test burden for study participants. The KIDSCREEN-27 is a psychometrically robust version of the original KIDSCREEN-52 version tested on 22827 children with a 69% response rate, assessing children and adolescents’ QoL [14]. The instrument has been found to have excellent cross-cultural comparative validity [3]. The 27-item questionnaire reflects 5 health-related QoL dimensions: Physical wellbeing (5 items), Psychological wellbeing (7 items), Parent relations and autonomy (7 items), Social support and peers (4 items) and School (4 items). Each item is scored on a 5-point scale (1 =“not at all”, 2 =“a little”, 3 =“moderately”, 4 =“much” and 5 =“very much”). Certain items are reversed when scoring the questionnaire. For each dimension, a scoring algorithm is used to calculate T-scores scaled with a mean of 50 and a standard deviation of 10 [17], while the total KIDSCREEN score is generated by summing up all item responses. Higher scores indicate better QoL [17]. In this study, the Swedish-language self-report and parental versions of the KIDSCREEN-27 were used [12].

#### Parental QoL (WHOQOL-BREF)

Parents’ QoL was assessed using WHOQOL-BREF questionnaire [18], with 26 items, where 24 of the items generate information about four domains: Physical health (7 items), Psychological health (6 items), Social relationships (3 items) and Environment (8 items). The remaining two items are global assessment questions on satisfaction with life quality and health. All items are scored on a 5-point scale. Domain scores are the sum of items; item scores are reversed wherever they indicate better QoL [19].

#### Sociodemographic factors

Sociodemographic factors measured in the study included country of birth, the child’s living status, (living with both parents, or living with either one of the parents or with other adults); the parent’s living status (living with or without a partner); employment (currently employed: permanent, temporary and self-employment; other: sick leave, parental leave, disabled, retired, student, job seeker, homeworker, and other); and educational level, measured as number of education years completed (≤11, 12–14, ≥15).

### Statistical Analysis

Normative data are presented based on child self-report as well as parent ratings. For child norms, all child self-report data were used (n = 202). For parent rating norms, and child-parent agreement analyses, single child and single parent respondents were excluded from the analysis, leaving the 175 child-parent pairs. The sample source is indicated in each table.

#### Child-report and parent ratings

We tested gender and age differences for both parent ratings and child-reported KIDSCREEN via separate analyses using *t-*tests and the Mann-Whitney *U*-test. All comparative analyses were based on two-tailed tests with a significance level of α = 0.05. Post-hoc Dunn tests were conducted for pairwise comparisons when the p-value for specific subscales was 0.05 or lower [20]. We also calculated floor and ceiling effects for each subscale; i.e., the proportion of parental ratings or children reporting the lowest and highest possible scores, respectively.

#### Child-parent agreement

We tested child-parent agreement in three ways: item-by-item, by dimensions and by total scores. Item-by-item child-parent agreement was tested for each KIDSCREEN item using the Prevalence- and Bias-Adjusted Kappa (PABAK) coefficient for ordinal data (PABAK-OS, a modified PABAK) [21]. In this case the ordinal data consisted of the response alternative to each item. Regarding dimensions and total KIDSCREEN scores, we wanted to facilitate case-finding for clinicians, and elected to follow a procedure similar to that used for the SDQ (for more details, see our SDQ article [22]). Binary cut-offs for child-reported and parent-rated dimension and total scores were thus defined as the lower 10% percentile based on the sample distribution. This is in line with the Eurobarometer study which showed a proportion of 11.6% among children across 27 countries with noticeably low overall wellbeing [5]. We then tested agreement for the dichotomized sub-scale and total scores using the PABAK. Total, continuous scores were evaluated using Bland-Altman plots [23], with the Y-axis representing differences in child-parent agreement (children’s score minus parent’s score), and the X-axis representing an average of the children’s and parental scores. The child-parent differences are expressed in medians (5%-95% inter-percentile range).

#### Sociodemographic factors and parental quality of life

Associations between sociodemographic factors and parental QoL were explored in relation to child self-reported and parent-rated KIDSCREEN scores, as well as for child-parent agreement (i.e. difference between self-reported and parent-rated total KIDSCREEN-27 score), using the Mann-Whitney *U*-test for parental gender, parent and child country of birth, living status, and parental employment. Exact tests were performed when the number of observations was less than ten in each category. The Kruskal-Wallis test was performed for parental educational level, and Spearman correlations were calculated for child and parent ages in relation to WHOQOL-BREF domains. We first analyzed the absolute value of the agreement, and later stratified by whether a child or his/her parent reported higher score.

Statistical analyses were performed using SAS 9.3 (SAS Institute INC. Cary, NC, USA), R software (http://www.r-project.org), and a web-based PABAK-OS calculator (http://www.singlecaseresearch.org/calculators/pabak-os).

## Results

### Child-reported KIDSCREEN scores

Table 3 presents child-reported KIDSCREEN-27 normative scores stratified by gender. Boys and girls did not differ significantly on KIDSCREEN-27 dimensions or total scores, but boys did show tendencies to score higher on the Psychological wellbeing (p = 0.06), Parent relations and autonomy (p = 0.09), and School environment (p = 0.09) dimensions.

**Table 3:** Child-reported T-scores for KIDSCREEN-27 subscales and total raw scores (range 27–135) by gender.

Kidscreen-27 T-score (SD)	Total Mean (SD)	Floor effect (%)	Ceiling effect (%)	Boys mean (SD)	Girls mean (SD)	p-value	missing
Physical well-being	48.8(9.21)	0	2.06	49.0 (8.31)	48.7 (9.78)	0.78^a^	8
Psychological well-being	53.4 (10.93)	0	11.86	55.2 (9.74)	52.2(11.51)	0.06^a^	8
Parent relations & autonomy	55.1 (9.88)	0	13.02	56.6 (10.27)	54.1 (9.52)	0.09^a^	10
Social support and peers	54.1 (8.17)	0	17.53	53.1(7.93)	54.7(8.29)	0.19^a^	8
School environment	55.8 (9.60)	0	18.13	57.2 (9.63)	54.8 (9.51)	0.09^a^	9
Total median raw score (25%-75% percentile)	115 (105–121)	-	-	116 (108–122)	114 (101.5–120.5)	0.16^b^	15
Total observations	N = 202			N = 78	N = 120		4

a. t-test.

b. Mann-Whitney U-test.

KIDSCREEN-27 child-reported normative scores stratified by age groups are presented in Table 4. Significant differences were identified in total scores as well as in every dimension except for Social support and peers. Post hoc analysis showed that, compared to children in the 12-year group, those in the 15- and 16–17 year groups scored lower in Physical wellbeing (p = 0.02 and 0.05 respectively) and 16–17 year olds scored lower on Psychological wellbeing compared to 12-year olds (p<0.001). On Parent relations and autonomy, children in the 16–17 year group scored lower than 13-year olds (p<0.001) and 15-year olds (p = 0.03). With regard to the total KIDSCREEN-27 score, children in the 12- and 13-year groups showed higher scores, indicating higher wellbeing than 16–17 year olds (*p*<0.05).

**Table 4:** Child-reported T-scores for KIDSCREEN-27 subscales and total raw score (range 27–135) by age groups.

Kidscreen-27 T-score (SD)	11y Mean (SD)	12yMean (SD)	13y Mean (SD)	14y Mean (SD)	15y Mean (SD)	16–17y Mean (SD)	p-value^a^
Physical well-being	48.9 (6.37)	52.5 (7.90)	51.4(9.58)	48.7 (9.56)	45.7 (7.42)	46.3 (11.15)	0.01*
Psychological well-being	54.9 (8.72)	59.5(10.85)	54.6(12.40)	52.5 (11.40)	51.8(9.31)	48.6 (9.38)	0.001*
Parent relations & autonomy	53.7 (7.31)	57.5(11.18)	58.0 (10.73)	52.6 (8.96)	58.0 (10.20)	51.3 (8.42)	0.01*
Social support and peers	53.8 (6.78)	56.2 (7.82)	55.4 (9.22)	54.0 (7.76)	53.9 (8.35)	51.8 (8.26)	0.20
School environment	58.0 (9.90)	59.0 (8.19)	57.3 (11.24)	51.5 (6.71)	54.4 (9.71)	54.3 (9.29)	0.01*
*Total median raw score (25%-75% percentile)*	116 (108–119)	122 (115–125)	119 (107–124)	114 (102–118)	113 (105–119)	108 (97–117)	0.001*
Total observations	N = 29	N = 31	N = 37	N = 25	N = 33	N = 43	

a. Kruskal-Wallis test.

### Parent-rated KIDSCREEN scores

Parent-rated scores, shown in Table 5 by gender and Table 6 by age, did not show any differences by gender or age.

**Table 5:** Parent-rated T-scores for KIDSCREEN-27 subscales and total raw scores (range 27–135) by gender.

Kidscreen-27 T-score (SD)	Total Mean (SD)	Floor effect %	Ceiling effect %	Boys Mean (SD)	Girls Mean (SD)	p-value	missing
Physical well-being	44.8 (7.34)	0	0	44.1 (7.05)	45.3 (7.51)	0.31^a^	6
Psychological well-being	52.3 (11.73)	0	6.63	51.5 (10.75)	52.7 (12.29)	0.50^a^	4
Parent relations & autonomy	54.7 (10.14)	0	6.15	54.7 (9.69)	54.6 (10.43)	0.94^a^	6
Social support and peers	52.8 (10.60)	1.53	7.14	51.9 (11.67)	53.3 (9.96)	0.42^a^	5
School environment	55.1 (9.34)	0	11.73	54.2 (8.83)	55.6 (9.63)	0.34^a^	5
Total median raw score (25%-75% percentile)	108 (100–116)	-	-	106 (99–116)	109 (100–117)	0.25^b^	10
Total observations	N = 175	-	-	N = 65	N = 110	-	0

a. *t*-test.

b. Mann-Whitney U-test.

**Table 6:** Parent-rated T-scores for KIDSCREEN-27 subscales and total raw score (range 27–135) by age groups.

Kidscreen-27 T-score (SD)	11y Mean (SD)	12y Mean (SD)	13y Mean (SD)	14y Mean (SD)	15y Mean (SD)	16–17yMean (SD)	p-value^a^
Physical well-being	46.8 (6.91)	44.6 (8.49)	46.0 (6.78)	45.6 (7.02)	42.1 (7.50)	44.5 (7.24)	0.27
Psychological well-being	51.9 (8.00)	52.6(13.41)	50.6 (12.26)	54.0 (13.61)	51.3 (11.39)	53.5 (11.94)	0.92
Parent relations & autonomy	55.4 (11.08)	52.3 (12.03)	52.6 (6.83)	54.4 (10.37)	55.2 (10.85)	54.8 (10.45)	0.96
Social support and peers	53.2 (11.32)	53.7 (7.72)	51.4 (11.87)	52.6 (11.12)	52.7 (12.78)	53.3 (8.42)	0.98
School environment	55.9 (8.61)	55.8 (9.08)	54.0 (8.14)	56.7 (9.62)	54.8 (11.9)	54.5 (8.77)	0.92
*Total median raw score(25%-75% percentile)*	110 (104–115)	108 (99–118)	106 (104–114)	105 (99.5–120.5)	104.5 (99–116)	110.5 (100–116)	0.95
Total observations	N = 25	N = 24	N = 33	N = 22	N = 31	N = 40	

a. Kruskal-Wallis test.

### Child-parent agreement on the KIDSCREEN-27

Child-parent agreement for the KIDSCREEN-27 items was as follows: 14 items (51.9%) had slight agreement, 12 (44.4%) had fair agreement, and one (3.7%) had agreement less than chance. Items in the Social support and peer relations dimension showed relatively low concordance, with PABAK-OS ranging from 0.13 to 0.31. However, substantial agreement was observed in the total dimension score analysis. See Table 7 for exact figures.

**Table 7:** Child-parent agreement^a^ on the KIDSCREEN-27 questionnaire.

Kidscreen-27 items	P0^b^	PABAK (95% CI)	p-value	Rating^c^
**Physical well-being**	0.88	0.75 (0.64–0.85)	**<0.05**	substantial
General health status	0.31	0.13(0.07, 0.19)	**0.001**	slight
Feel fit and well	0.43	0.28 (0.22, 0.34)	**0.000**	fair
Physically active	0.30	0.13 (0.07, 0.19)	**0.001**	slight
Run well	0.28	0.10 (0.04, 0.16)	**0.011**	slight
Feel energetic	0.35	0.19 (0.13, 0.25)	**0.000**	slight
**Psychological well-being**	0.81	0.63 (0.51–0.75)	**<0.05**	substantial
Life been enjoyable	0.18	-0.02 (-0.08, 0.04)	0.590	less than chance
In good mood	0.43	0.28 (0.22, 0.34)	**0.000**	fair
Have had fun	0.49	0.31 (0.25, 0.38)	**0.000**	fair
Feel sad	0.29	0.11 (0.05, 0.17)	**0.003**	slight
Feel too sad and don’t want to do anything	0.38	0.23 (0.17, 0.29)	**0.000**	fair
Feel lonely	0.43	0.29 (0.23, 0.35)	**0.000**	fair
Be happy with the way you are	0.29	0.11 (0.05, 0.17)	**0.006**	slight
**Parent relations & autonomy**	0.85	0.69 (0.58–0.80)	**<0.05**	substantial
Have enough time for yourself	0.40	0.26 (0.20, 0.32)	**0.000**	fair
Be able to do things you want to do in free time	0.35	0.19 (0.13, 0.25)	**0.000**	slight
Parent(s)have enough time for you	0.33	0.16 (0.10, 0.22)	**0.000**	slight
Parent(s) treat you fairly	0.36	0.20 (0.14, 0.26)	**0.000**	slight
Be able to talk to your parents when you wanted to	0.34	0.34 (0.12, 0.24)	**0.000**	fair
Have enough money to do the same things as friends	0.41	0.26 (0.20, 0.32)	**0.000**	fair
Have enough money for expenses	0.34	0.18 (0.11, 0.23)	**0.000**	slight
**Social support and peers**	0.85	0.70 (0.59–0.81)	**<0.05**	substantial
Spend time with friends	0.32	0.15 (0.09, 0.21)	**0.000**	slight
Have fun with friends	0.45	0.31 (0.25, 0.37)	**0.000**	fair
Helped each other with friends	0.33	0.16 (0.10, 0.22)	**0.000**	slight
Rely on friends	0.31	0.13 (0.07, 0.19)	**0.001**	slight
**School environment**	0.82	0.65 (0.53–0.76)	**<0.05**	substantial
Be happy at school	0.46	0.33 (0.27, 0.39)	**0.000**	fair
Get on well at school	0.38	0.22 (0.16, 0.28)	**0.000**	fair
Be able to pay attention	0.36	0.14 (0.07, 0.21)	**0.000**	slight
Get along well with teachers	0.42	0.23 (0.16, 0.30)	**0.000**	fair
**Total Kidscreen-27 score**	0.82	0.64 (0.52, 0.76)	**<0.05**	substantial

a. Individual item responses for children and parents were compared based on raw scores. Domain and Total Kidscreen-27 score child-parent comparisons were based on dichotomous variables, see method.

b. Proportion of agreement.

c. PABAK<0, agreement less than chance; 0.01≤ PABAK≤ 0.2, slight agreement; 0.21≤ PABAK≤0.40, fair agreement; 0.41≤ PABAK≤0.60, moderate agreement; 0.61≤ PABAK≤0.80, substantial agreement; 0.81≤PABAK≤0.99, almost perfect agreement [24].

**Fig 1:**
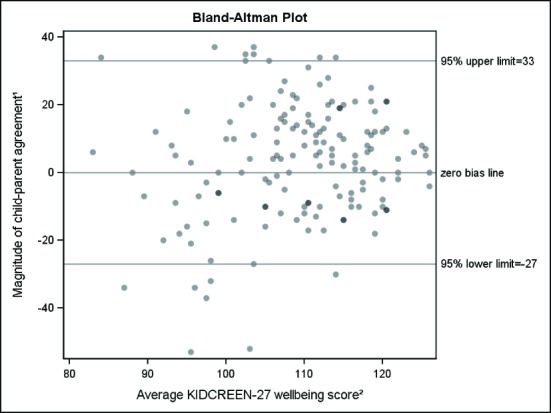
Bland-Altman plot for child-parent agreement differences on the total KIDSCREEN-27 score. 1. Subtracting the parental scores (Ps) from the children’s scores (Cs); Cs-Ps. 2. Average of the children’s (Cs) and parental (Ps) scores [(Cs + Ps)/2]. Higher average scores indicate greater wellbeing.

To further examine child-parent agreement on the total KIDSCREEN-27 score, Bland-Altman plots were generated by plotting the relationship between differences and means, where child-parent agreement differences are indicated by dots in relation to the zero bias line on the *y-*axis, and the shading of the dot darkens with increasing numbers of child-parent pairs with the same difference score (see Fig 1). The median (5%-95% percentile) for child-parent difference in total KIDSCREEN-27 score was 5 (-27, 33), and children also rated themselves higher than their parents, as indicated by the larger proportion of dots above the zero bias line in comparison to the proportion of dots below it in Fig 1. Visual interpretation of the Bland-Altman plot suggests that when the average wellbeing score is lower (*x-*axis), parents seem to rate their children as having relatively higher total wellbeing, as shown by the relatively larger proportion of cases *below* the zero bias line. On the other hand, as the average wellbeing score increases, parents tend to rate their children as having relatively lower total wellbeing scores, as indicated by the relatively larger proportion of cases *above* the zero bias line.

### Sociodemographic and parental QoL correlates

Crude associations were calculated between sociodemographic factors and parental QoL on the one hand and self-reported and parent-rated KIDSCREEN-27 total scores (S1 Table).

#### Child-reported scores

Children who lived with both parents reported significantly higher wellbeing according to the KIDSCREEN, in comparison to children who lived with either parent or with other adult constellations (*p* = 0.01). A tendency towards an association between parental education and children’s wellbeing was observed, where children with parents with 11 years or less education reported higher wellbeing according to the KIDSCREEN, compared to children with higher educated parents (*p* = 0.08). Parental QoL did not generally show significant associations with children’s wellbeing, with *r* coefficients around zero. A small association was found for physical QoL, however, where higher parental physical QoL was associated with lower child-reported wellbeing (*r* = -0.17, *p* = 0.03).

#### Parent ratings

Parents of children who live with both parents rated their children higher on wellbeing, as expressed in the total KIDSCREEN score. All the parental QoL subscales were positively correlated with parent-rated KIDSCREEN-27 scores. Parents with higher quality of life thus also rated increased wellbeing for their children.

#### Child-parent agreement

We explored associations between sociodemographic factors and parental QoL and the absolute *size* of agreement as well as the *direction* of child-parent agreement. No significant associations were found between sociodemographic factors or parental QoL and size or direction of child-parent *dis*agreement (S2 Table).

## Discussion

This study provided random population sample data on Swedish pre-adolescent and adolescent self-reported and parent-rated wellbeing, as measured by the KIDSCREEN-27. The study also reported data on child-parent agreement, as well as sociodemographic associations. In general, we found that older children reported lower wellbeing than younger children. No self-reported gender differences were found. Child-parent agreement was generally high when differentiating between ordinary and noticeably low wellbeing. Children who lived with both parents had higher wellbeing than those who did not, according to both child self-reports and parent ratings. In addition, parents with higher QoL reported higher wellbeing for their children. Sociodemographic factors and parental QoL were not associated with agreement between child self-reports and parent ratings.

### Levels of wellbeing

The median child self-reported raw score for KIDSCREEN-27 for the present sample was 115. In comparison to the European norms reported by Ravens-Sieberer et al. (2013), the children in our sample scored slightly lower on Physical wellbeing (T-score 48.8 in our study compared to 49.94 on the European norms), but higher on all other four KIDSCREEN-27 dimensions: Psychological wellbeing (53.4/49.77), Parent relations and autonomy (55.1/49.99), Social Support and peers (54.1/49.94) and School (55.8/50.01) [3].

We found child self-reported differences by age on all dimensions except for Social support and peers. Older children’s wellbeing was significantly lower than that of younger children. These findings concord with differences between European 11-, 13- and 15-year olds, where child self-reported wellbeing has been found to decrease with age [25] as well as indications of the same phenomenon among all Swedish schoolchildren aged 13 and 16 [13]. This finding is not surprising given the critical developmental nature of the period between pre-adolescence and early adulthood [26]. In our study, parent-rated wellbeing showed no age differences, in contrast to child-reported wellbeing. Parent ratings for children 6 to 17 in the 27-country Eurobarometer study have, however, indicated lower wellbeing for 11–17 year olds in comparison to 6–10 year olds, where 15–17 year olds had progressively lower wellbeing than 11–14 year olds on the KIDSCREEN-10 [5]. The sample size for the latter study was 12 783 and the level of wellbeing varied widely between countries. Parent ratings for Sweden have not been reported separately in prior published research, but our findings suggest that while such ratings may serve as a complementary proxy assessment of children’s wellbeing, making sure to take children’s own reports into account can be of utmost importance to more accurately assess children’s wellbeing.

We found no significant gender differences, child self-reported or parent-rated, on the KIDSCREEN-27 dimensions and total score, but boys did show tendencies to report higher scores than girls on Psychological wellbeing, Parent relations and autonomy and School. Earlier studies with larger samples have found more extensive gender differences [12, 13], with boys reporting higher wellbeing than girls on all dimensions except for Social support and peers, and we conjecture that the lack of significant gender differences in our study could be due to the relatively small sample size.

### Child-parent agreement

Item-by-item child-parent agreement generally varied from slight to fair. However, using dichotomous variables that generate a clear differentiation between ordinary and noticeably low wellbeing indicated substantial child-parent agreement both for the five KIDSCREEN-27 dimensions and for the total KIDSCREEN raw score and the PABAK-OS. Bland-Altman plots, based on the total KIDSCREEN-27 raw score as a continuous variable, showed that children reported slightly higher wellbeing in comparison to parental ratings, especially when the child/parent average wellbeing score was high.

Earlier research examining KIDSCREEN child-parent agreement mainly used paired-t-tests and intra class correlation coefficients (ICC), with inconsistent findings [27–29]. We evaluated child-parent agreement using Bland-Altman plots and the kappa statistic, methods recommended for examining inter-rater agreement for continuous and categorical variables respectively [24, 30]; in contrast, ICCs can sometimes be viewed as measures of reliability rather than agreement [31]. Nonetheless, our finding of good to excellent child-parent agreement on KIDSCREEN dimensions was in line with the majority of previous studies [27, 28]. In contrast, a study from Spain showed low to moderate agreement in a random census-based sample of children 8–18 years old [29]. The inconsistency of the results in these studies may be due to heterogeneity in the study populations, as suggested by earlier KIDSCREEN group findings that indicated country and age influences on child-parent agreement in different directions [32]. Our results also showed that looking at wellbeing as a continuous variable resulted in lower agreement than when dichotomizing the outcome to discern those with noticeably low wellbeing.

### Sociodemographic correlates

Living with both parents was associated with higher child-reported and parent-rated wellbeing, and parents with high QoL rated their children’s wellbeing higher. This is in line with recent Swedish findings that children living with both parents have fewer everyday difficulties than children in single care or living in joint physical custody arrangements; furthermore, children of parents with higher life satisfaction were found to experience fewer difficulties [33]. Parental education was not significantly related to children’s wellbeing. Research on the relationship between parental education and children’s wellbeing in Europe has shown an unclear picture, at least when wellbeing is measured with the KIDSCREEN-10 [34]. Lower family affluence, reflecting financial resources, has, however, been found to be associated with lower child wellbeing [5, 25, 34]. Our finding of a non-significant tendency to lower wellbeing among families with higher education adds to prior inconsistent findings on the relationship between education and wellbeing. Further analysis of possible associations between specific dimensions of wellbeing and education might yield a more nuanced picture, but this is a question we did not explore due to reasons of space.

### Strengths and limitations

This study contributes normative data for child-reported and parent-rated wellbeing by gender and age, as well as child-parent agreement data, from a randomized Swedish population sample. To our knowledge, this is the first study using PABAK-OS, a Kappa index not influenced by bias or prevalence [21], for assessing child-parent agreement based on dichotomous variables reflecting wellbeing on the five KIDSCREEN-27 dimensions as well as the total wellbeing score.

We have described study limitations for this data sample in our separate article on the SDQ [22], and summarize the limitations briefly here. Our sample response rate was relatively low, but typical for population studies conducted via postal mail; furthermore, we view the rate as acceptable in view of our need for child-parent paired responses for assessing child-parent agreement. Nonetheless, the study would have had better power if we had been able to recruit more respondents, particularly child-parent pairs. This would have been useful in relation to the non-significant tendencies we identified towards differences between boys and girls on the KIDSCREEN-27, as well as the tendency toward lower wellbeing among more highly educated parents. Furthermore, we were unable to assess the possible confounding influence of sociodemographic variables for the effects of age and gender on children’s QoL, although this is not a problem of great magnitude given the lack of associations between most sociodemographic variables and QoL (see S1 and S2 Tables). In terms of representativity, a larger sample might have improved gender representativity, as this sample includes a larger proportion of females than males, to some extent among children and to a large extent among the parents. Also, we had an overrepresentation of parents with higher education (15 years or more) than in the total population and of Swedish-born parents and children. However, in terms of employment, our employment rate of 86% matched the 85% rate for the total Swedish population [35], and parental quality of life was similar to levels in the Norwegian and Danish population samples where the WHOQOL-Bref has been studied [36]. A lack of representativeness may have affected the levels of KIDSCREEN-27 scores such that wellbeing is overestimated. However, insufficient representativeness is not believed to markedly affect the associations between gender, age and socioeconomic status on the one hand, and wellbeing as measured by the KIDSCREEN-27[37]. Finally, we collected data for only one parent and asked no questions about the other parent, with the consequence that socio-demographic variables may have been misclassified, for example if one parent was employed and the other not. This could lead to an underestimation of our results regarding sociodemographic correlates.

## Conclusions

This study presents normative data for Swedish children’s wellbeing, in a format that can be used to compare individual children’s wellbeing to population levels. The study thus makes a significant contribution to the literature on children’s mental health and wellbeing in Sweden. We found age differences in child reports of wellbeing but not in parental ratings, and we also found that parents with higher QoL rated their children’s QoL higher, an association not reflected in children’s self-reports which were unaffected by parental QoL. The study thus suggests the importance of taking children’s own reports into consideration and not relying solely on parental or other informant ratings. However, our relatively small sample and single occasion design limit the wide applicability of our findings. Future research on children’s wellbeing and mental health status should be conducted at regular intervals, and encompass larger samples in a cross-sectional design. This could contribute to eventually minimizing the gap between mental health needs and services, to help more children and adolescents grow up to be healthy, mature and happy adults [1, 26].

## Supporting Information

S1 Table Crude associations of sociodemographic factors with child-reported and parent-rated KIDSCREEN total scores. (DOCX)

S2 Table Sociodemographic factors and child-parent agreement on Kidscreen-27 total score. S2 Table legend. a. Child-reported score minus parent-rated score is greater than 0, meaning that the child feels better than the parent thinks s/he does.
b. Child-reported score minus parent-rated score is equal to or less than 0, meaning that the child feels the same or worse than the parent thinks s/he does.
c. Mann-Whitney U-test; d. Kruskal-Wallis test; e. Spearman correlation; f. exact test was performed when the number of observation fewer than 10 in any of the categories.
(DOCX)
