# Supplementary material for: Children’s Quality of Life Based on the KIDSCREEN-27: Child Self-Report, Parent Ratings and Child-Parent Agreement in a Swedish Random Population Sample
Source: PLoS One. 2016 Mar 9;11(3):e0150545. doi: 10.1371/journal.pone.0150545 (PMC4784934; doi:10.1371/journal.pone.0150545)
Supplement: S1 Table — (DOCX) [file pone.0150545.s001.docx]

|  | **Child-reported total score** |  | **Parent-rated total score** |  |
| --- | --- | --- | --- | --- |
|  | **Median (25%, 75%)** | **p-value** | **Median (25%, 75%)** | **p-value** |
| **Parent Sex^a^** |  |  |  | 0.90 |
| Males | 115 (104, 121) | 0.89 | 108.5 (102-115) |  |
| Females | 115 (103, 120) |  | 107(99-117) |  |
| **Child born in Sweden ^a^** |  | 0.91 |  | 0.86 |
| Yes | 115 (105, 121) |  | 108 (100-116) |  |
| No | 115 (97, 121) |  | 105.5 (93.0-122.0) |  |
| **Parent born in Sweden^a^** |  | 0.66 |  | 0.64 |
| Yes | 115 (104, 120) |  | 107 (100-116) |  |
| No | 116 (105, 122) |  | 110.5 (104-118) |  |
| **Child living status^a^** |  | **0.01** |  | **0.03** |
| with both parents | 116 (106-122) |  | 109 (102-117) |  |
| with either parents/others | 112 (96-118) |  | 104 (92-114) |  |
| **Parent living status ^a^** |  | 0.23 |  | 0.08 |
| with partner | 117 (108, 122) |  | 109 (102-116) |  |
| without partner | 115 (105, 120) |  | 104 (95.0-112.5) |  |
| **Employment^a^** |  | 0.24 |  | 0.59 |
| Employed | 115 (104, 120) |  | 107 (100-116) |  |
| Other | 118 (105, 124) |  | 109 (95-116) |  |
| **Parent Education^b^** |  | 0.08 |  | 0.55 |
| 11 years or less | 118 (106-124) |  | 109 (99-118) |  |
| 12-14 years | 113 (99-120) |  | 106 (99-115) |  |
| 15 years or more | 114 (104-119) |  | 109.5 (102-116) |  |
| **Parent age** | *r coefficient* | *P-value* | *r coefficient* | *P-value* |
|  | 0.05 | 0.51 | 0.01 | 0.86 |
| **WHOQOL-BREF^c^** |  |  |  |  |
| Physical Health | -0.17 | **0.03** | 0.34 | **0.0001** |
| Psychological Health | -0.09 | 0.28 | 0.43 | **0.0001** |
| Social relationships | 0.02 | 0.76 | 0.29 | **0.0001** |
| Environment | -0.08 | 0.30 | 0.42 | **0.0001** |
