# Supplementary material for: Children’s Quality of Life Based on the KIDSCREEN-27: Child Self-Report, Parent Ratings and Child-Parent Agreement in a Swedish Random Population Sample
Source: PLoS One. 2016 Mar 9;11(3):e0150545. doi: 10.1371/journal.pone.0150545 (PMC4784934; doi:10.1371/journal.pone.0150545)
Supplement: S2 Table — legend. a. Child-reported score minus parent-rated score is greater than 0, meaning that the child feels better than the parent thinks s/he does. b. Child-reported score minus parent-rated score is equal to or less than 0, meaning that the child feels the same or worse than the parent thinks s/he does. c. Mann-Whitney U-test; d. Kruskal-Wallis test; e. Spearman correlation; f. exact test was performed when the number of observation fewer than 10 in any of the categories. (DOCX) [file pone.0150545.s002.docx]

|  | **KIDSCREEN total score disagreement**  **absolute value** |  | **KIDSCREEN total score: Child self-reported score greater than parent-rated^a^** |  | **KIDSCREEN total score: Child self-reported score equal to or less than parent-rated score^b^** |  |
| --- | --- | --- | --- | --- | --- | --- |
|  | **Median (25%, 75%)** | **p** | **Median (25%, 75%) (n)** | **p** | **Median (25%, 75%) (n)** | **p** |
| **Child Sex^c^** |  | 0.85 |  | 0.31 |  | 0.60 |
| Boys (N=57/65) | 12 (5, 18) |  | 12 (5, 19) (38) |  | -14 (-17, -5) (19) |  |
| Girls(N=98/110) | 11 (6, 21) |  | 13 (8, 21) (53) |  | -10 (-14, -4) (45) |  |
| **Parent Sex^c^** |  | 0.96 |  | 0.67 |  | 0.88 |
| Males (N=31/35) | 10 (7, 19) |  | 11 (8, 18) (21) |  | -10 (-21, -2) (10) |  |
| Females (N=124/140) | 12 (5, 19.5) |  | 12.5 (6, 21) (70) |  | -10 (-15, -5) (54) |  |
| **Child born Sweden ^c^** |  | 0.29 |  | 0.37^f^ |  | 0.60 |
| Yes (N=147/167) | 8 (4, 14.5) |  | 12.5 (7, 21) (86) |  | -10 (-16, -5)(61) |  |
| No (N=8/8) | 12 (6, 20) |  | 8 (5, 15) (5) |  | -8 (-14, -2) (3) |  |
| **Parent born Sweden^c^** |  | 0.12 |  | 0.16 |  | 0.41^f^ |
| Yes (N=137 /156) | 12 (6, 20) |  | 13 (8,21)(80) |  | -10 (-16, -5)(57) |  |
| No (N=18/19) | 7 (2, 18) |  | 7(2, 19)(11) |  | -7 (-17,0) (7) |  |
| **Child living status^c^** |  | 0.36 |  | 0.76 |  | 0.12 |
| with both parents ( N=125/141) | 11 (6, 18) |  | 12 (7,21)(73) |  | -9.2 (-14, -4)(52) |  |
| with either parents/others (N=30/34) | 13.5 (6, 20) |  | 13.5 (6, 19)(18) |  | -14 (-23.5, -9.5)(12) |  |
| **Parent living status ^c^** |  | 0.96 |  | 0.70 |  | 0.34 |
| with partner (N=132/150) | 12 (6, 19) |  | 12 (7, 20)(75) |  | -10 (-16, -5)(57) |  |
| without partner (N=23/25) | 11 (4, 27) |  | 13 (5.5, 29) (16) |  | -9 (-14, 0) (7) |  |
| **Employment^c^** |  | 0.57 |  | 0.37 |  | 0.76 |
| Employed (N=137/152) | 11 (6, 19) |  | 12 (7, 20)(79) |  | -10 (-17, -4)(58) |  |
| Other (N=18/22) | 12.5 (6, 21) |  | 18.5 (8.5, 22.5) (12) |  | -11 (-13, -5)(6) |  |
| **Parent Education^d^** |  | 0.95 |  | 0.83 |  | 0.74 |
| 11 years or less ( N=38/44) | 12.5 (5, 20) |  | 15 (6, 21)(27) |  | -9 (-14, -2) (11) |  |
| 12-14 years ( N=52/60) | 11 (6.5, 18) |  | 12.5 (9, 19.5)(28) |  | -8.5 (-17.5, -3.5)(24) |  |
| 15 years or more ( N=65/70) | 11 (6, 18) |  | 12.0 (6, 20.5)(36) |  | -10 (-16, -6)(29) |  |
|  | *r-coefficient* |  | *r-coefficient* |  | *r-coefficient* |  |
| **Child age^e^** | 0.05 | 0.49 | 0.08 | 0.44 | -0.12 | 0.33 |
| **Parent age^e^** | -0.10 | 0.21 | -0.03 | 0.78 | 0.22 | 0.08 |
| **WHOQOL-BREF^e^** | *r-coefficient* |  |  |  |  |  |
| Physical Health | -0.02 | 0.80 | -0.01 | 0.89 | -0.11 | 0.36 |
| Psychological Health | 0.11 | 0.19 | -0.09 | 0.40 | -0.003 | 0.98 |
| Social relationships | -0.01 | 0.84 | 0.06 | 0.57 | -0.01 | 0.94 |
| Environment | -0.08 | 0.35 | -0.09 | 0.38 | -0.09 | 0.48 |

a. Child-reported score minus parent-rated score is greater than 0, meaning that the child feels better than the parent thinks s/he does.

b. Child-reported score minus parent-rated score is equal to or less than 0, meaning that the child feels the same or worse than the parent thinks s/he does.

c. Mann-Whitney U-test; d. Kruskal-Wallis test; e. Spearman correlation; f. exact test was performed when the number of observation fewer than 10 in any of the categories.
